# Raised Plasma Neurofilament Light Protein Levels After Rewarming Are Associated With Adverse Neurodevelopmental Outcomes in Newborns After Therapeutic Hypothermia

**DOI:** 10.3389/fneur.2020.562510

**Published:** 2020-10-23

**Authors:** Divyen K. Shah, Ping K. Yip, Akif Barlas, Pavithira Tharmapoopathy, Vennila Ponnusamy, Adina T. Michael-Titus, Philippa Chisholm

**Affiliations:** ^1^The Royal London Hospital, Barts Health NHS Trust, London, United Kingdom; ^2^The Centre for Neuroscience, Surgery and Trauma, Barts and The London School of Medicine and Dentistry, Blizard Institute, Queen Mary University of London, London, United Kingdom; ^3^Centre for Genomics and Child Health, Barts and The London School of Medicine and Dentistry, Blizard Institute, Queen Mary University of London, London, United Kingdom; ^4^Ashford and St. Peter's Hospitals NHS Foundation Trust, Chertsey, United Kingdom; ^5^Homerton University Hospitals NHS Foundation Trust, London, United Kingdom

**Keywords:** newborn, hypoxic-ischemic encephalopathy, neurodevelopment, neurofilament light protein, therapautic hypothermia

## Abstract

**Aim:** To determine the predictive value of plasma neurofilament light protein (NfL) as a prognostic marker for outcomes in babies who have undergone therapeutic hypothermia (TH) for hypoxic ischemic encephalopathy (HIE).

**Method:** NfL levels from three groups of term newborns were compared: (1) those with mild HIE who did not receive TH, (2) newborns treated with TH who had minimal or no brain injury on MRI, and (3) newborns treated with TH who had substantial brain injury on MRI. Follow-up outcomes were collected from 18 months onward.

**Results:** Follow-up was available for 33/37 (89%) of children. A cutoff NfL level >436 pg/ml after rewarming (median age 98 h) was associated with adverse outcome with a diagnostic sensitivity 75%, specificity 77%, PPV 75%, and NPV 77%. NfL levels at earlier time points were not predictive of outcome.

**Interpretation:** This pilot study shows that persistently raised plasma NfL levels after rewarming are associated with adverse outcomes in babies with HIE who have undergone TH.

## Introduction

Mild therapeutic hypothermia (TH) has been shown to be effective in improving outcomes in newborns suffering hypoxic–ischemic encephalopathy (HIE) with a number needed to treat of 7–9 (1) and is a recognized standard of care (2). At present, there are no specific blood biomarkers in clinical use that assist in stratification of severity of encephalopathy and selection for neuroprotection.

Neurofilament light (NfL) is a structural protein present in myelinated axons and is abundant in the central nervous system (CNS). In response to CNS axonal damage

caused by inflammatory, neurodegenerative, traumatic, or vascular injury, the release of NfL sharply increases (3). The NfL that is released reaches the interstitial fluid, which communicates freely with the CSF, and the blood, where its concentration is roughly 40-fold lower than it is in the CSF. Increased concentrations of NfL in blood have been shown in patients with chronic neurodegenerative conditions such as Alzheimer's disease (4) amyotrophic lateral sclerosis (4, 5) and multiple sclerosis (6). As such, the potential role of NfL as a biomarker for brain injury in the context of HIE in newborns remains to be explored more fully.

Previously, we have shown that it is feasible to study NfL levels from newborns (7). We demonstrated that raised plasma NfL levels assessed at three time points from newborns undergoing TH for HIE were associated with cerebral MRI findings predictive of unfavorable outcomes. However, the relationship between plasma NfL and later outcome in this group of patients has yet to be demonstrated. We hypothesize that raised plasma NfL levels during the newborn period are predictive of longer-term adverse outcome in babies with HIE.

## Method

Term newborns with HIE were recruited between January 2014 and January 2016 from four tertiary neonatal centers as part of the Brain Injury Biomarkers in Newborns Study (BIBiNS): the Royal London Hospital, Homerton University Hospital, Ashford, and St Peter's Hospitals and Southampton University Hospital. This study was carried out with research ethics committee approval (REC reference: 13/LO/17380).

### Participants

These recruits have been described previously (7). For the purposes of the NfL study, samples were studied from three groups of newborns from this cohort: (1) consecutively recruited babies with mild acidosis and/or mild HIE (mild HIE group) who did not fulfill standard criteria (8) for TH and were managed conservatively; (2) consecutively recruited babies who underwent TH but had cerebral MRI predictive of adverse outcome as described previously (9) (TH unfavorable MRI group), and (3) consecutively recruited babies who underwent TH but had cerebral MRI predictive of a favorable outcome (TH favorable MRI group).

### Blood Sampling

Newborns who had TH for HIE had blood samples taken at three time points: (i) after the infant had reached target temperature (S1), (ii) prior to commencing rewarming (S2), and (iii) after completing rewarming (S3). Infants with mild HIE who did not receive cooling therapy had a single specimen taken (S1). Preparation and analysis of plasma samples has been previously described (7).

### Outcomes and Neurodevelopment Assessment

Neurodevelopmental testing was administered by the local centers and were collected as per local follow-up protocols for babies with HIE, as previously described (9). Abnormal outcome was defined as a cognitive or motor composite score <85 (−1 SD) in either cognitive or motor domains, a diagnosis of cerebral palsy or death related to associated causes.

### Statistical Analysis

Analysis was carried out using SPSS V25.0 (IBM Corp, Armonk, New York) and GraphPad Prism V7 (GraphPad Software, La Jolla California USA). Continuous variables between groups were compared using Mann–Whitney U and categorical variables using x^2^ in SPSS. NfL levels for S1 were positively skewed and thus were log transformed prior to analysis. A receiver operating characteristic (ROC) curve was created in GraphPad Prism, comparing the mild HIE group with TH babies with abnormal outcome. Additionally, TH babies with abnormal outcomes were compared with the TH babies with normal outcomes at timepoints S1, S2, and S3. Cutoff levels for the ROC curves were obtained where sensitivity and specificity were the highest, where a value *p* < 0.05 was highly significant.

## Results

Eleven babies were studied in the mild HIE group, 13 in the TH favorable MRI group, and 13 in the TH unfavorable MRI group. The perinatal characteristics of these three groups have already been described in Table 1 of our previous publication (7). Perinatal characteristics of babies with mild HIE compared with all the newborns treated with TH combined are shown in Table 1 in the present publication. Babies who received TH were more likely to have chest compressions, resuscitation with cardiac medication, meconium aspiration, positive blood culture, seizures, anticonvulsants, and use of inotropes (*p* < 0.05).

**Table 1:** Perinatal characteristics of the mild HIE group compared with all the TH babies in the study.

	**Mild No TH**	**Mod–Severe TH**	***p*-value**
*n*	11	26	
Male	5/11	18/26	0.189
Birth weight (g)	3,360 (2,971, 3,987)	3,493 (2,935, 3,800)	0.233
10-min Apgar	9 (8, 9)	5 (4, 7)	0.316
Chest compressions	0/9	10/21	0.023^*^
Resuscitation with cardiac drugs	0/9	6/25	0.030^*^
Worst pH in first hour	6.96 (6.91,7.03)	6.89 (6.72, 7.00)	0.096
Worst base deficit in first hour	−15 (−12, −8)	−13.4 (−18.0, −8.4)	0.096
Maternal pyrexia	2/10	0/25	0.482
Chorioamnionitis	0/9	0/24	0.602
Sentinel event	1/11	7/26	0.255
Meconium aspiration	0/10	3/26	0.012^*^
Blood glucose (<2.6 mmol/l)	0/8	0/17	0.298
Positive blood culture	0/10	1/26	0.009^*^
Clinical seizures	0/10	20/26	0.000^*^
Anticonvulsants given	0/10	18/26	0.005^*^
Inotropes used	0/11	11/26	0.000^*^

* *Denotes significance where p < 0.05. Values with brackets are median (interquartile range)*.

### NfL Levels

NfL levels in the three groups have previously been described (7). The single blood sample from the mild HIE group was obtained at a median age of 24 h. In the cooled babies' samples, S1, S2, and S3 were obtained at median ages of 18, 54, and 98 h, respectively. Only 3/8 babies in the mild HIE group who had outcomes available had a detectable plasma NfL level (83, 156, and 172 pg/ml). Of these three, one child who had high functioning autism with difficulties with social interaction was assigned an abnormal outcome.

### Outcomes

Outcomes were available for 33/37 (89%) children, with neurodevelopmental assessment performed at a median age of 2.7 [interquartile range (IQR) 2.0, 3.2] years. Of the 33 babies, 23 had Bayley-III assessments, four had ASQ-3 (Ages and Stages Questionnaire−3) assessments, and six were assigned outcomes by DS based on the follow-up information supplied by the local team. Of the six children with assigned outcomes, three were from the mild HIE group and three had received TH. Only one of the six was in the abnormal outcome category; a baby from the mild HIE group who was diagnosed with features of high functioning autism.

Three children in the mild HIE groups were lost to follow-up, and one child in the TH favorable MRI group was not available, detailed as below.

Outcomes were available for 8/11 children in the mild HIE group. Seven of these children had normal outcomes. One child, who had high-functioning autism with difficulties with social interaction, was assigned an abnormal outcome. Outcomes were available for 12/13 in the TH favorable MRI group. One child was discharged from follow-up at 5 months as he was thought to be doing well, and no further follow-up data was available. Of the other 12, 11 children had a normal outcome and one had an abnormal outcome. This child had normal motor findings but severe impairment in cognition and language in keeping with other features of autism. Outcomes were available for all 13 children in the TH unfavorable MRI group. Of these, two had normal and 11 abnormal outcomes. Of these 11, nine had a diagnosis of cerebral palsy.

### Diagnostic Accuracy of Plasma NfL Levels in Prediction of Outcomes

**Figure 1:**
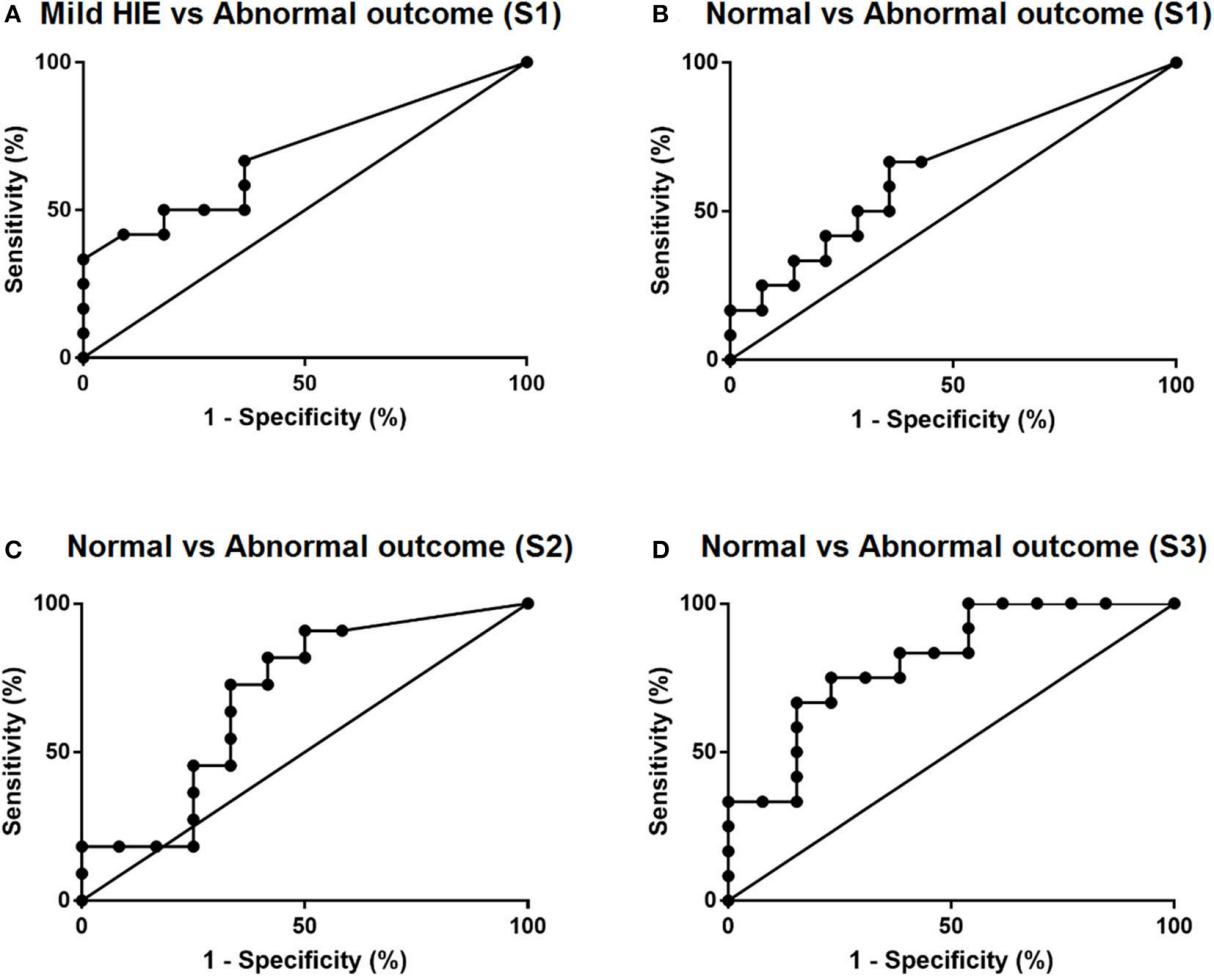
ROC curves of plasma NfL levels. **(A)** Comparison of mild HIE babies vs. cooled babies with abnormal outcomes and **(B–D)** comparison of cooled babies with normal and abnormal outcomes at timepoints S1, S2, and S3 respectively.

To assess the diagnostic value of plasma NfL levels in predicting later neurodevelopment, ROC curves were constructed, along with cutoff levels of plasma NfL for each sampling time (Figure 1). The area under the curve (AUC) for each blood sample showed that plasma NfL is a variable classifier of neurodevelopmental outcome (Table 2). A cutoff of 436 pg/ml at timepoint S3 was strongly predictive of later neurodevelopmental outcome with an AUC 0.81, *p* = 0.009, sensitivity = 75%, and specificity = 77%. Of the nine children who developed cerebral palsy, all nine had detectable NfL levels and seven had levels >436 pg/ml (Table 3).

**Table 2:** The predictive value of plasma NfL levels at timepoints S1, S2, and S3 with later neurodevelopment.

**Sample**	**Area under the curve**	**SE**	***p*-value**	**Cutoff (pg/ml)**	**Sensitivity (%)**	**Specificity (%)**	**PPV (%)**	**NPV (%)**
S1	0.654	0.104	0.156	28	67	63	53	75
S2	0.686	0.115	0.132	166	73	67	67	73
S3	0.808	0.087	0.009	436	75	77	75	77

*SE, standard error; PPV, positive predictive value; NPV, negative predictive value*.

**Table 3:** Characteristics of babies with cerebral palsy.

**Study number**	**Apgar 10 min**	**Respiratory support 10 Min**	**Chest compressions**	**Worst pH in first hour**	**Worst base deficit in first hour**	**Sentinel event**	**Clinical seizure (Y/N)**	**PLIC**	**BGT**	**WM**	**Cortex**	**MRI outcome**	**S1**	**S2**	**S3**	**CP GMFCS**
1	4	Y	Y	6.8	−18	Y	N	2	3	3	3	U	29.394	224.709	674.385	5
2	4	Y	N	6.94	−19.8	N	Y	2	3	3	3	U	0	188.718	1,625.672	5
3	0		Y	7.3		N	Y	2	3	1	1	U	69.941	422.949	1,013.869	3
4	5	Y	Y	6.63	−25.7	N	Y	2	3	2	0	U	2,506.037	2,255.24	4,697.766	5
5		Y	N	6.64	−22	N	Y	2	3	2	1	U	172.529	158.548	334.448	2
6		Y	Y	6.6	−27	N	Y	1	2	1	2	U	0		563.196	5
7	9	Y	N	7	−16.4	N	Y	2	2	3	0	U	148.327	172.168	151.207	4
8	10	N	Y	6.77	−19.7	N	Y	2	2	1	0	U	0	0	511.332	4
9	1	Y	-	6.9	−25.5	N	Y	0	2	3	2	U	412.535	381.27	843.895	3

*Y, yes; N, no; U, unfavorable MRI outcome; SE, standard error; PLIC, posterior limb of internal capsule; BGT, basal ganglia and thalami; WM, white matter; CP GMFCS, cerebral palsy gross motor function classification system*.

***MRI scores noted in****Table 3****as adapted from Rutherford et al***.

***Posterior limb of internal capsule on T1***.

*0, normal; 1, reduced or asymmetrical signal intensity; 2, severe injury with reversed or abnormal signal intensity bilaterally on T1- and/or T2-weighted images*.

***Basal ganglia and thalami***.

*0, normal; 1, mild injury (focal abnormal signal intensity); 2, moderate injury (multifocal abnormal signal intensity); 3,indicates severe injury (widespread abnormal signal intensity)*.

***White matter***.

*0, normal; 1, mild injury (long T1 and T2 in periventricular white matter only); 2, long T1 and T2 in subcortical WM and or focal punctate lesions or focal infarction; 3, severe widespread abnormalities including long T1 and T2 +/– infarction +/– hemorrhage*.

***Cortical involvement***.

*0, normal; 1, mild (1–2 sites cortical highlighting/decreased T1); 2,moderate (3 sites involved); 3, severe (more than three sites)*.

## Discussion

In this paper, we provide additional data to complement our previous work in which we have demonstrated the feasibility of using NfL levels as a biomarker of brain injury in term newborns who have undergone TH. We demonstrate that a cutoff level >436 pg/ml after rewarming was associated with abnormal outcome with a diagnostic sensitivity 75%, sensitivity 77%, PPV 75%, and NPV 77%.

Thus far, this is the first study to examine the relationship between serial plasma NfL levels in newborns undergoing TH and later neurodevelopmental outcome in newborns. Toorell et al. (10) highlighted the importance of NfL as an early predictive marker in asphyxia from cord blood. Our study comparing levels from babies with mild HIE who are not cooled with those from cooled babies with substantial brain injury on MRI as well as babies with relatively normal cerebral MR imaging shows that plasma NfL levels rise during the period of TH (7), and the present work shows that NfL levels after the rewarming period, taken at days 4–5 (median age of 98 h in this group), are predictive of later outcomes.

Various novel blood biomarkers of brain injury have been investigated in newborns with HIE (11); however, as yet none has been established in clinical practice.

We chose to explore NfL levels as researchers at our institute, and others have found levels raised in other neurological conditions (5, 12, 13). In cases of severe traumatic brain injury (TBI) in adults, plasma NfL levels were shown to correlate with 12-month follow-up outcomes with an AUC of 0.70, with an AUC of 0.99 at admission to detect TBI. Rodent studies in mild TBI have shown that NfL levels are robustly detectable in younger rodents; however, plasma NfL levels were markedly reduced in older rodents, making NfL a potential biomarker in the younger population (14). Mattsson et al. showed that high plasma NfL levels correlated with poor cognition and Alzheimer's disease-related brain atrophy, with an AUC of 0.87 (15). In comparison to these conditions, the brain injury observed in newborn HIE is arguably a less heterogeneous entity than TBI; is thought to be associated with an acute generalized hypoxic–ischemic insult to a relatively immature brain; and follows a shorter disease time course than conditions such as Alzheimer's disease. We speculate that these differences may account for the difference in NfL cutoff levels that we note in the babies compared to these other groups.

Our work has a number of limitations. As a pilot feasibility study, the numbers are small and the study is not adequately powered to detect smaller differences. Also, we do not have NfL levels from cord blood or earlier samples from babies prior to commencing TH. As the samples are obtained once TH is already established, the role of TH in attenuating NfL levels cannot be excluded. Of the three sequential samples studied, as only NfL levels from the last sample (obtained after rewarming) at a median age of 98 h are significantly associated with outcome, we postulate that NfL levels are unlikely to provide an early biomarker for selection of newborns for neuroprotection but may assist with identifying infants who will benefit from additional treatments in the post-acute period, as they become available. A larger cohort study is required with serial sampling commencing at earlier time points.

## Conclusion

We have demonstrated in this pilot study that it is feasible to study plasma NfL as a biomarker of brain injury in newborns and that levels after rewarming correlate with later outcomes in newborns who have undergone TH for moderate to severe HIE. As such, NfL may be a candidate biomarker for neuroprotective interventions in the post-acute period, once these are available. A larger prospective cohort study is required.

## Data Availability Statement

The raw data supporting the conclusions of this article will be made available by the authors, without undue reservation.

## Ethics Statement

The studies involving human participants were reviewed and approved by Bromley Research Ethics Committee. Written informed consent to participate in this study was provided by the participants' legal guardian/next of kin.

## Author Contributions

DS conceptualized and designed the study, designed the data collection sheets, recruited patients for the study, performed the data analysis, drafted the initial manuscript, approved of the final manuscript as submitted, and the principal investigator, had full access to all the data in the study and takes responsibility for the integrity of the data and the accuracy of the data analysis. PY conceptualized and designed the study, performed the laboratory analysis, assisted with the data analysis, assisted in preparing the manuscript, and approved the final manuscript. AB coordinated the neurodevelopmental follow-up, assisted in preparing the manuscript, and approved the final manuscript. PT performed the data analysis, assisted in preparing the manuscript, and approved the final manuscript. VP recruited patients for the study, assisted in preparing the manuscript, and approved the final manuscript. AM-T provided intellectual input, assisted in preparing the manuscript, and approved the final manuscript. PC coordinated the neurodevelopmental follow-up, provided intellectual input, assisted in preparing the manuscript, and approved the final manuscript. All authors contributed to the article and approved the submitted version.

## Conflict of Interest

The authors declare that the research was conducted in the absence of any commercial or financial relationships that could be construed as a potential conflict of interest.

## Abbreviations

NfL: neurofilament light protein
TH: therapeutic hypothermia.
